# *In Vivo* Confocal Microscopy and Anterior Segment Optical Coherence Tomography Findings in Two Cases with Mucopolysaccharidoses

**DOI:** 10.4274/tjo.galenos.2020.53503

**Published:** 2020-06-27

**Authors:** Yalçın Karaküçük, Banu Bozkurt, Merve Şahin, Süleyman Okudan

**Affiliations:** 1 Selçuk University Faculty of Medicine, Department of Ophthalmology, Konya, Turkey

**Keywords:** In vivo confocal microscopy, mucopolysaccharidoses, Morquio syndrome, Scheie syndrome

## Abstract

The mucopolysaccharidoses are a group of disorders caused by inherited defects in lysosomal enzymes resulting in widespread intracellular and extracellular accumulation of glycosaminoglycans. Due to the mucopolysaccharidoses subtype, glycosaminoglycans can be deposited in many organs and tissues including cornea. In this report, we presented *in vivo* confocal microscopy and anterior segment optical coherence tomography findings in a 39-year old man with Scheie syndrome and a 41-year old woman with Morquio syndrome (Heidelberg Retina Tomograph 3 Rostock module, Germany) and reviewed the literature. On *in vivo* confocal microscopy, there were multiple small and larger hyperreflective deposits in the epithelium, Bowman layer and anterior stroma and abnormally shaped, elongated keratocytes with hyporeflective round structures, which might be vacuoles in the anterior-mid stroma. In anterior segment optical coherence tomography images, accumulation of glycosaminoglycans deposits lead to an increased hypereflective appearance throughout the thickened cornea.

## Introduction

The mucopolysaccharidoses (MPSs) are a group of disorders caused by inherited defects in lysosomal enzymes resulting in widespread intracellular and extracellular accumulation of glycosaminoglycans (GAGs). MPSs are subdivided according to their enzyme defects and their systemic manifestations.^1^

Scheie syndrome (MPS-IS; 607016) is a rare form of MPSs with an autosomal recessive pattern. It is caused by mutations in the *IDUA *gene (4p16.3) that lead to a partial deficiency in the α-L-iduronidase enzyme and lysosomal accumulation of dermatan sulphate and heparan sulphate. In MPS-IS, ocular findings have been reported including corneal clouding, retinal pigment epithelial changes, acute angle closure glaucoma, optic nerve swelling and atrophy, and changes similar to macular edema as well as systemic findings.^2^ Corneal clouding becomes visually significant after the first decade of life in MPS-IS. Diagnosis is based on the detection of increased urinary secretion of heparan and dermatan sulfate through the 1.9-dimethylmethylene blue test, the electrophoresis of GAGs, enzymatic deficiency in leukocytes or fibroblasts and genetic testing.^1,2,3^ In MPS-IS, the main histopathological features are vacuoles containing fibrillogranular material in corneal epithelial cells and keratocytes, a disrupted epithelial basement membrane, slightly attenuated Bowman layer, changes in collagen fibers and keratocytes with GAG accumulation, a normal Descemet’s membrane, and inconstant vacuoles in the endothelial cells.^4^

Morquio syndrome (MPS-IVA; 253000) is an autosomal recessive lysosomal storage disorder caused by mutations in *GALNS *(16q24.3) and deficient N-acetylgalactosamine-6-sulphatase. This enzyme deficiency leads to progressive accumulation of keratan sulphate and chondroitin-6-sulphate in the cornea, bone, cartilage, and ligaments. In addition to systemic findings, the ocular findings in patients with MPS-IVA include cataract, optic atrophy, tapetoretinal pigmentary degeneration, and corneal clouding.^5^ Corneal clouding and retinopathy, which are common in all variants of MPS, are the major causes of impaired vision. On light microscopy, the basal cells of the epithelium are swollen and the keratocytes and endothelium contain numerous intracytoplasmic bodies.^6^ At the ultrastructural level, the apical portion of the basal cells is packed with small clear membrane-bound vacuoles which decrease in numbers in the wing cell and superficial cell layers.^6^ Keratocytes include intracytoplasmic inclusions in the form of multilaminar bodies, fingerprint whorl patterns, fibrillogranular inclusions, small lipid vacuoles, and clear vacuoles. The endothelial cell cytoplasm also contains membrane-bound vacuoles which fuse to form large empty cytoplasmic spaces, causing the cell membranes to collapse.

*In vivo* confocal microscopy (IVCM) of the living human cornea offers the ability to perform real-time imaging without tissue damage and has been widely used in clinical practice to evaluate corneal and ocular surface pathologies. In the literature, there are only a few studies about the use of IVCM and anterior segment optical coherence tomography (AS-OCT) in MPSs.^7,8,9,10,11,12^ Herein, we report the clinical, AS-OCT, and laser IVCM findings of 2 patients with MPS-IS and MPS-IVA. Our aim was to compare our findings with previous studies in the literature, determine whether these imaging devices can show the typical histopathological features of MPS, and demonstrate the differences between MPS-IS and MPS-4A.

## Case Reports

### Case 1

**Figure 1:**
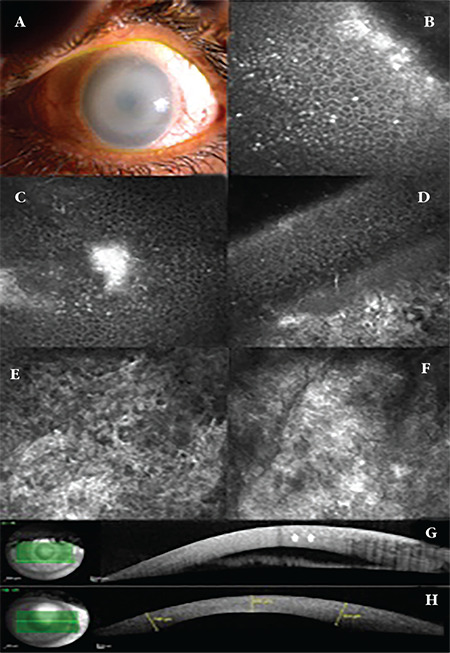
Slit-lamp biomicroscopy of a patient with mucopolysaccharidose-IS demonstrating severe corneal clouding obscuring iris details (a). *In vivo* confocal microscopy images showing multiple small hyperreflective deposits in the epithelium (b), larger opacities in the epithelium, Bowman layer, and anterior stroma extending up to 80 µm in length (c,d), and hyperreflective anterior and mid stroma (e,f). Anterior segment optical coherence tomography images showing thickened corneas with increased hyperreflectivity in the stroma, especially in a granular pattern in the anterior stroma (arrow) (g,h)

A 39-year-old man with a prior diagnosis of MPS-IS who had been using a dorzolamide-timolol fixed combination and brimonidine for the last 5 years presented to our clinic for refractive error and glaucoma evaluation. He had normal intellectual function, an abnormal gait, and skeletal dysmorphism. He had low levels of leukocyte α-L-iduronidase enzyme activity. His visual acuity was 4/20 in the right eye (OD) with correction of +8.50/+1.00x130 diopter (D) and 4/20 in the left eye (OS) with +8.50/+1.25x5 D. Biomicroscopic examination showed multiple corneal opacities in the corneal stroma and severe corneal clouding (Figure 1a). Intraocular pressure (IOP) measured with a Goldmann applanation tonometer was 24 mmHg in both eyes (OU). The fundus could not be examined due to severe corneal haze. Laser IVCM (Heidelberg Retina Tomograph 3 Rostock module, Heidelberg Engineering GmbH, Heidelberg, Germany) showed multiple small hyperreflective deposits in the epithelium (Figure 1b), larger opacities (up to 80 µm in length) in the epithelium, Bowman layer and anterior stroma (Figure 1c, d), hyperreflective corneal stroma with round hyporeflective structures inside (Figure 1e, f). The posterior stroma and the endothelium could not be assessed due to stromal hyperreflectivity. On AS-OCT (Spectralis®, Heidelberg Engineering, Heidelberg, Germany) examination, thick corneas (central corneal thickness [CT] 658 µm OD and 664 µm OS, peripheral CT 950 µm) with increased hyperreflectivity in the stroma were observed (Figure 1g). Iridocorneal measurements could not be taken. Anterior chamber depth measured by a corneal topography device with a Scheimpflug camera (Sirius, CSO, Italy) were 3.55 mm OD and 3.43 mm OS.

### Case 2

**Figure 2:**
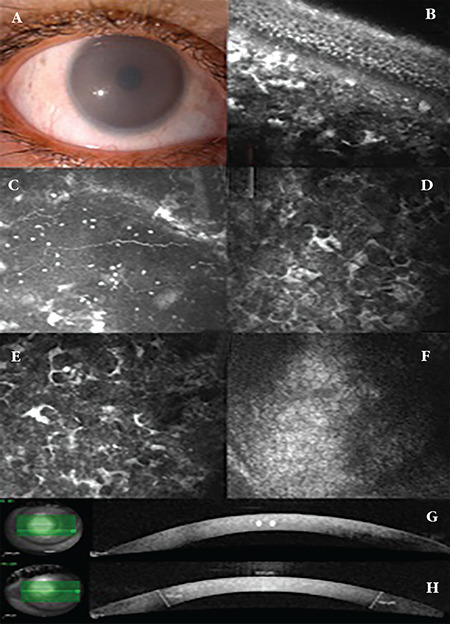
Slit-lamp biomicroscopy of a patient with mucopolysaccharidose-IVA demonstrating moderate corneal clouding (a). *In vivo* confocal microscopy imaging revealed hyperreflective basal epithelial cells, dot-like and larger opacities extending 25-30 µm in length in the Bowman layer and anterior stroma (b) and around the subepithelial nerve plexus (c). Abnormally shaped, elongated keratocytes and dark, round structures outlined by white borders, which might be vacuoles, lacunae, and microdots were observed in the anterior-mid stroma (d,e), while the endothelial layer, although not very clear, seemed normal (f). Anterior segment optical coherence tomography images, showing thickened corneas with diffuse hyperreflectivity in the stroma, especially in a granular pattern in the anterior stroma (arrow) (g,h)

A 41-year-old woman with a diagnosis of MPS-IVA had been followed-up for glaucoma and using timolol maleate-brimonidine fixed combination for the last 3 years. She had low levels of leukocyte N-acetylgalactosamine-6-sulphatase enzyme activity, severe skeletal deformities and gait disturbance, but no intellectual impairment. Bilateral best-corrected visual acuity was 16/20 with a correction of +0.50/+1.00x135 D OD and +1.00x105 D OS. IOP was 22 mmHg OU. Slit-lamp biomicroscopic examination revealed moderate corneal clouding and diffuse stromal opacities (Figure 2a). The fundus examination could hardly be done, but the optic discs seemed normal with a cup/disc (C/D) ratio of 0.4. IVCM revealed hyperreflective basal epithelial cells, dot-like and larger opacities (up to 25-30 µm in length) in the Bowman layer and the anterior stroma (Figure 2b) and around the subepithelial nerve plexus (Figure 2c). Abnormally shaped, elongated keratocytes and hyporeflective dark, round structures outlined by white borders (which might be vacuoles), lacunae, and microdots were observed in the anterior-mid stroma (Figure 2d-e). The endothelial layer, although not very clear, seemed normal (Figure 2f). In AS-OCT images, diffuse hyperreflectivity in the corneal stroma and thick corneas were noted (CCT 634 µm OD and 629 µm OS) (Figure 2g). Iridocorneal angle measurements, which could be taken only from patient 2, revealed that the angle opening distance at 500 µm from the scleral spur (AOD 500) was 210 µm on the temporal side and 251 µm on the nasal side. The temporal and nasal iridocorneal angles were 21.7° and 25.9°, respectively. Anterior chamber depth measured by a corneal topography device with a Scheimpflug camera were 3.53 mm OD and 3.67 mm OS.

## Discussion

In AS-OCT imaging, the internal structure of the corneas of both patients showed increased hyperreflectivity. The thickness measurements of the cornea were high, around 650 µm in the center and 950 µm in the periphery. Ahmed et al.^7^ reported a narrower and more compact anterior segment and angle configuration in MPS VI than in MPS I via Visante AS-OCT. MPSs type I and type VI had a greater tendency to corneal clouding, and cornea was thicker compared to type II. They also reported that corneal thickening in the periphery and narrowing of the iridocorneal angle might be the mechanisms of raised IOP in patients with MPS I and VI. Aragona et al.^9^ showed diffuse hyperreflective structures throughout the thickened cornea in a patient with MPS-IS using OCT. In our patient with MPS-IVA, temporal and nasal AOD 500 were much lower than cut-off values for narrow angles (320 µm and 340 µm, respectively).

Grupcheva et al.^8^ were the first to report findings using a real-time, slit-scanning IVCM to examine a 13-year-old boy with MPS-IS. There were bright cells in the basal epithelium and the keratocytes exhibited markedly altered morphology, often being round or elliptical in shape, with clearly demarcated, hyporeflective centers. The sub-basal nerve plexus contained corneal nerves similar in thickness and density to those in a normal cornea, although the nerve fibers were irregular and somewhat difficult to distinguish, possibly due to underlying fibrosis. Endothelial cell count was normal for the age group, but the cells exhibited mild polymegethism. Aragona et al.^9^ described the IVCM, OCT, and histological findings of 2 corneas from a 25-year-old man with MPS-IS. Laser IVCM showed diffuse or granular hyperreflectivity in the basal epithelial cells. The keratocytes were highly reflective, causing a web-shaped stromal appearance, while the endothelial cells were barely visible. Our MPS-IS patient was a 39-year-old man who had severe corneal clouding, low visual acuity, and glaucoma. Laser IVCM revealed hyperreflective deposits in the basal epithelium, Bowman layer, and anterior stroma, which were more demonstrative than the previous studies.^8,9^ This might be explained by the older age of our patient and continuous accumulation of GAG over years. In advanced cases, deposits and fibrosis lead to increased hyperreflectivity, which obscures the detailed examination of the cellular structures in the stroma and endothelium by IVCM. In our case, we could not detect typical keratocyte vacuolation and the endothelium changes.

Stewart et al.^10^ reported laser confocal findings of a 7-year-old boy with MPS-IVA who had very mild diffuse, fine granular corneal stromal appearance and good visual acuity. IVCM demonstrated a diffuse hyperreflectivity immediately posterior to the Bowman’s layer and in the anterior and middle stroma. The keratocyte cytoplasm was clearly visible; it had a fine, granular appearance, and the nuclei were rounded and exhibited vacuolation, particularly in the posterior stroma. The corneal epithelium, subbasal nerves, and endothelium appeared normal.

Our MPS-IVA patient was a 41-year-old woman who had been followed-up for corneal clouding and glaucoma for the last 3 years. In IVCM imaging, there were hyperreflective basal epithelial cells, dot-like and larger opacities around the subepithelial nerve plexus, in the Bowman layer and the anterior stroma, which were not reported by Stewart et al.^10^ In MPSs, GAGs accumulate progressively with age, which might explain why deposits were not observed in the corneal epithelium and around the subbasal nerves in the 7-year-old child reported by Stewart et al.^10^ Also, there were abnormally shaped, elongated keratocytes and dark, round structures outlined by white borders, which were believed to be vacuoles within the keratocytes, lacunae, and microdots in the anterior-mid stroma. The endothelial layer, although not very clear, seemed normal.

IVCM and AS-OCT imaging technologies can be used to show macroscopic and microscopic corneal changes related to MPSs. In this study, using AS-OCT, we found that accumulation of GAG deposits lead to an increased hypereflective appearance throughout the thickened cornea. IVCM showed hyperreflective dot-like and larger deposits in the basal epithelium, Bowman layer, and stroma and altered morphology of keratocytes with vacuoles inside. In advanced cases with severe corneal clouding, examination of the stroma and endothelium with IVCM is not possible due to increased reflectivity in the anterior stroma.
